# The ORF3 Protein of Genotype 1 Hepatitis E Virus Suppresses TLR3-induced NF-κB Signaling via TRADD and RIP1

**DOI:** 10.1038/srep27597

**Published:** 2016-06-08

**Authors:** Man He, Min Wang, Ying Huang, Wenju Peng, Zizheng Zheng, Ningshao Xia, Jian Xu, Deying Tian

**Affiliations:** 1 Department of Gastroenterology, Tongji Hospital, Tongji Medical College, Huazhong University of Science and Technology, Wuhan, 430030, China; 2 National Institute of Diagnostics and Vaccine Development in Infectious Disease, School of Public Health, Xiamen University, Xiamen, 361005, China; 3 Department of Infectious Disease, the Central Hospital of Fuling District, Chongqing, 404100, China

## Abstract

Hepatitis E virus (HEV) genotype 1 infection is common and can emerge as outbreaks in developing areas, thus posing a threat to public health. However, due to the absence of feasible animal models, the mechanism of HE pathogenesis remains obscure. The HEV pathogenic mechanism has been suggested to be mediated by the immune system and not by direct viral duplication. We firstly discovered that the open reading frame 3 (ORF3) protein of genotype 1 HEV downregulates TLR3-mediated NF-κB signaling in Human A549 Lung Epithelial Cells (A549 cells) which were exposed to different TLR agonists associated with viral nucleic acids. Additionally, we identified the P2 domain of ORF3 as being responsible for this inhibition. Intriguingly, tumor necrosis factor receptor 1-associated death domain protein (TRADD) expression and receptor-interacting protein kinase 1 (RIP1) K63-ubiquitination were reduced in the presence of both ORF3 and Poly(I:C). Furthermore, we found that Lys377 of RIP1 acts as the functional ubiquitination site for ORF3-associated inhibition. Overall, we found that ORF3 protein downregulates TLR3-mediated NF-κB signaling via TRADD and RIP1. Our findings provide a new perspective on the cellular response in HEV infection and expand our understanding of the molecular mechanisms of HEV pathogenesis in innate immunity.

Epidemics of HEV are increasingly being reported^1^. Although the disease is often sporadic and self-limited, HEV infection, may also lead to acute hepatitis or, in rare cases, fulminant hepatic failure. High mortality rates of approximately 20% in pregnant women during the second and third trimesters are also observed with HEV infection^2^. The virus, which belongs to the genus *Orthohepevirus* of the family *Hepeviridae*^3^, is a small, nonenveloped virus (27–34 nm in diameter) with a linear, single-stranded, plus-sense RNA genome of 7.2 kilobases^4^ consisting of three open reading frames: ORF1, ORF2 and ORF3^5^. ORF1 encodes a nonstructural polyprotein involved in HEV replication^6^, and ORF2 encodes the capsid protein that is responsible for virion assembly and immunogenicity^7^. A multifunctional phosphoprotein that links intracellular transduction pathways, reduces the host inflammatory response and protects virus-infected cells is encoded by ORF3^8^. The N-terminal half of ORF3 protein contains two hydrophobic domains, D1 and D2; the C-terminal half contains two proline-rich domains, P1 and P2^9^. The virus can be classified into four distinct genotypes, a single known serotype. Genotypes 1 and 2 are transmitted exclusively in humans, and genotypes 3 and 4 are zoonotic^10^. HEV genotype 1 has been implicated in large epidemics in all endemic countries, including the 1978–1979 epidemic in Kashmir, India^11^, and the 1986–1988 epidemic in the Xinjiang Uyghur autonomous region, China^12^. In addition, HEV genotype 1 is responsible for the highest incidence of acute hepatitis in young adults, tends to lead to higher morbidity and mortality during pregnancy, and has a substantial rate of fulminant hepatic failure^13^. Recently, efficient culture systems for HEV have been established in A549 cells, which are derived from human lung cancer^14^. Our research team has found that in A549 cells, the ORF3 protein of genotype 1 HEV inhibits the NF-κB signaling induced by TNF-α^15^. However, the precise function of the ORF3 protein in other intrinsic signaling pathways remains unclear. Due to the absence of a feasible *in vivo* model, the purpose of our research was to verify the role of genotype 1 HEV ORF3 protein in TLR-induced NF-κB signaling at the cellular level.

Innate immunity has been described as the sentinel of the immune system^16^, and this arm comprises various germline-encoded pattern recognition receptors (PRRs) that detect microbial components known as pathogen-associated molecular patterns (PAMPs)^17^. TLRs were the first PRRs to be characterized, and nucleic acids derived from viruses act as agonists for multiple TLRs. For example, TLR3 detects double-stranded RNA (dsRNA)^18^, TLR7 and TLR8 recognize single-stranded RNA (ssRNA), and TLR9 senses unmethylated CpG DNA^19^. In unstimulated cells, these TLRs are located in the endoplasmic reticulum (ER); upon activation, they are translocated via the Golgi apparatus to endosomes, where they are processed by proteases to encounter the internalized nucleic acid ligands^20^,^21^. Individual TLRs recruit myeloid differentiation primary response 88 (Myd88) or TIR-domain-containing adaptor inducing interferon (IFN)-β (TRIF, also known as TICAM1) to initiate a specific response. MyD88 can transmit signals derived from all TLRs, except for TLR3, which utilizes the TRIF-dependent pathway^22^. RIG-I-like receptors (RLRs), including RIG-I, Mda5 and LGP2, recognize cytoplasmic viral RNA^23^. In response to viral genetic materials, IRF3/7-dependent production of IFN as well as NF-κB-dependent inflammatory cytokines and chemokines are induced to disrupt viral replication.

The transcription factor NF-κB regulates multiple physiological functions, including the immune response, protection against apoptosis, and inflammation^24^. The NF-κB family consists of RelA (P65), RelB, c-Rel, P50/p105 (NF-κB1) and p52/p100 (NF-κB2), which can form both homodimers and heterodimers^25^. The P65/P50 heterodimer is the most common activated form in TLR and RLR signaling^26^, whereas in resting cells, the P65/P50 heterodimer is sequestered in the cytoplasm by the inhibitor of NF-κB (IκB) protein. IκB phosphorylation by the IκB kinase (IKK) complex is induced by a stimulus, leading to K48 ubiquitination of IκB and subsequent proteasomal degradation. As a result, P65 is liberated and migrates to the nucleus, where it promotes the expression of target genes^27^. During this event, TRADD and RIP1 play a crucial role in the inhibition of ORF3, whereby TRADD acts as the connector and RIP1 as an executor that is ultimately responsible for signal broadcasting. Ubiquitination (Ub) is a significant physiological modification of a target protein that regulates signal transduction by all receptor systems. K48-linked Ub moieties are recognized by the proteasomal system for the degradation of modified proteins, whereas K63-linked Ub promotes protein-protein interactions and signaling^28^. In the present study, we demonstrated that the Lys63-branched polyubiquitination of RIP1 is indispensable to Poly(I:C)-induced NF-κB activation and that Lys^377^ of RIP1 was a crucial ubiquitination site for the inhibitory role of ORF3.

Our findings provide valuable information regarding the suppression of TLR3-mediated NF-κB signaling in A549 cells by the ORF3 protein of genotype 1 HEV via TRADD and RIP1. The objective of this research was to elucidate the function of the ORF3 product and to expand our understanding of the cellular processes of HEV pathogenesis.

## Results

### HEV ORF3 protein inhibited the Poly(I:C)-induced activation of NF-κB

**Figure 1:**
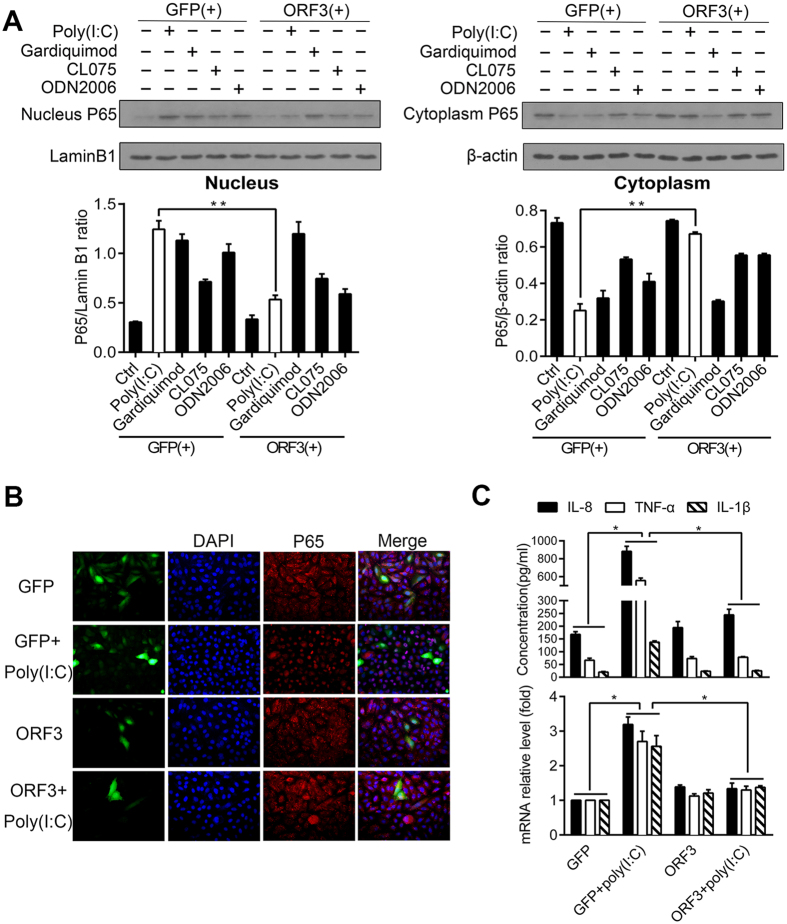
HEV ORF3 inhibits the Poly(I:C)-induced activation of NF-κB. (**A**) A549 cells were transfected with either ORF3 or GFP plasmid for 48 h, stimulated with different ligands for 12 h, and then processed for western blotting using an anti-P65 antibody. ORF3 protein suppressed the Poly(I:C)-induced nuclear translocation of P65 when A549 cells were stimulated with the optimal doses of different ligands. Densitometric analyses are represented in the bar graph. We quantified the gray scale ratio of the density of P65 bands in the nuclear/cytoplasmic fractions to the density of Lamin B1/β-actin bands. Significant differences (the blank bar) are denoted by asterisks (**P < 0.01). (**B**) Immunofluorescence assay for detecting the distribution of P65. A549 cells were transfected with either ORF3 or GFP plasmid (green) for 48 h and stimulated with Poly(I:C) (10 μg/ml) for 12 h. Red fluorescence indicates the detection of the P65 protein; blue shows nuclear DNA counterstaining with DAPI. The cells were observed by fluorescence microscopy (magnification: 400X). (**C**) The concentrations of NF-κB-responsive genes (IL-8, TNF-a and IL-1β) were measured in cell culture supernatants using ELISA (upper panel). The relative mRNA expression of IL-8, TNF-a and IL-1β were calculated by normalization to the housekeeping control using RT-PCR, and fold changes relative to the expression levels are presented (lower panel) (*P < 0.05). The data are displayed as the means ±SD of three independent experiments.

**Figure 2:**
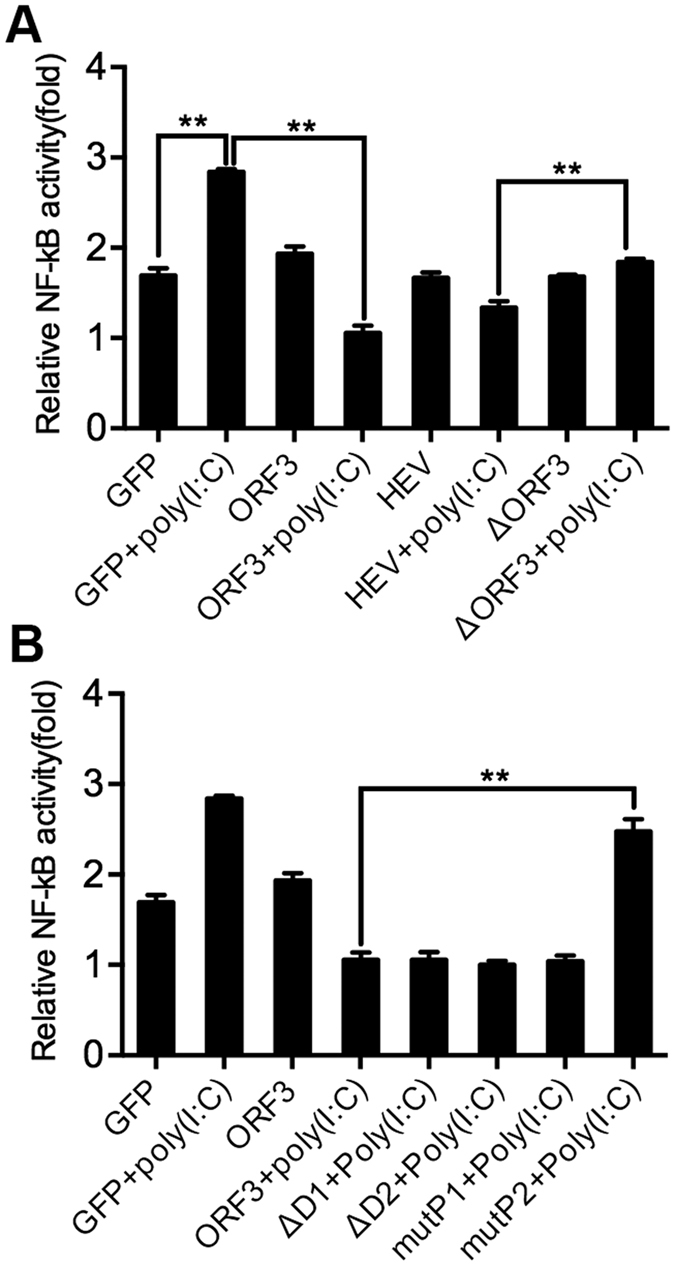
The P2 domain of HEV ORF3 displays an inhibitory role. A549 cells were co-transfected with 20 ng of pNF-κB-Luc (firefly luciferase), 5 ng of pRL-TK (Renilla luciferase) and the indicated amounts of expression plasmids. After 48 h, the cells were stimulated with or without Poly(I:C) for 12 h. Cell lysates were assayed for luciferase activity. To correct for transfection efficiency, the firefly luciferase values were normalized based on the Renilla luciferase values. The relative fold differences of luciferase activity from the control cells are quantified. (**A**) ΔORF3 recovered from Poly(I:C)-induced NF-κB activation. Induction of NF-κB activity by Poly(I:C) was suppressed in cells pretreated with pSK-Sar55 and ORF3, whereas NF-κB activation was observed in pretreated ΔORF3 cells. (**B**) The P2 domain of HEV ORF3 plays an inhibitory role in Poly(I:C)-induced NF-κB signaling. We constructed four plasmids with different ORF3 domain mutations. Stimulation with Poly(I:C) led to increased luciferase expression in mutP2 protein compared to ORF3-expressing cells. All results represent triplicate experiments. The data represent the average of three independent experiments (**P < 0.01).

To observe the role of the ORF3 protein in TLR signaling, we chose to examine TLR3, TLR7, TLR8 and TLR9, which are related to the detection of ssRNA viruses. We first assessed TLR expression in A549 cells (Figure S1A) by fluorescence microscopy and found that 15–20% of cells emitted green fluorescence when ORF3 was successfully transfected; we also confirmed ORF3 expression by western blotting (Figure S1B). We then used the corresponding ligand to stimulate TLR signaling, and a series of dose-dependent experiments was carried out to identify the optimal doses. As cognate ligands of agonists can stimulate NF-κB signaling, P65 nuclear translocation was used to assess activaton of NF-κB. Poly(I:C) (a TLR3 ligand), Gardiquimod (a TLR7 ligand), CL075 (a TLR8 ligand) and ODN2006 (a TLR9 ligand) had peak effects on NF-κB signaling at concentrations of 10 μg/ml, 3 μg/ml, 5 μg/ml, and 3 μM, respectively (Figure S2). We also detected P65 in the cytoplasm in the control and GFP groups. According to the gray scale ratio of the target protein and the loading control in the western blot analysis, ORF3 hindered the nuclear translocation of P65 after stimulation with Poly(I:C) (P < 0.01), which was not discovered with the other agonists (Fig. 1A). The distribution of P65 in the presence of ORF3 following Poly(I:C) stimulation was observed by immunofluorescence microscopy (Fig. 1B). The dual-luciferase reporter gene system showed that Poly(I:C) could induce NF-κB activity in the GFP group and that ORF3 alone had no effect on NF-κB activity. However, NF-κB activity was reduced in the ORF3 group in the presence of Poly(I:C) (Fig. 2A). Because the activation status of NF-κB can influence inflammatory cytokines, including IL-8, TNF-α and IL-1β, mRNA levels were measured using RT-PCR to illustrate the function of ORF3. Poly(I:C) promoted the mRNA expression of inflammatory factors in the GFP group (P < 0.05), whereas the mRNA levels of inflammatory factors were lower in the ORF3 with Poly(I:C) group compared to the GFP with Poly(I:C) group (P < 0.05). There were no significant differences between the ORF3 group and the ORF3 with Poly(I:C) group (P > 0.05) (Fig. 1C). The cellular supernatants of cells transfected with either GFP or ORF3 with or without Poly(I:C) were examined by ELISA, with similar findings (Fig. 1C).

### The P2 domain of HEV ORF3 plays an inhibitory role in Poly(I:C)-induced NF-κB signaling

Based on the above results, we evaluated whether pSK-Sar55 without ORF3 could diminish NF-κB activity. Thus, a western blot analysis was performed to identity infectious clones of pSK-Sar55 and ΔORF3 in A549 cells, to detect whether pSK-Sar55 could express the ORF2 and ORF3 proteins, and to ascertain whether ΔORF3 could express the ORF2 protein without expressing the ORF3 protein (Figure S3A). Cellular supernatants were tested for the HEV antigen by ELISA, and the OD values of the positive samples were higher than the cutoff value. On the basis of these values, PSK-Sar55 and ΔORF3 successfully produced the HEV. Similar results were observed for the PLC/PRF/5 cellular supernatant (Figure S3B). Induction of NF-κB activity by Poly(I:C) was suppressed in cells pretreated with pSK-Sar55 and ORF3, whereas NF-κB activation was observed in pretreated ΔORF3 cells (Fig. 2A).

To determine which segment of HEV ORF3 has a crucial role in the inhibitory effect, we constructed four plasmids with different ORF3 domain mutations. A dual-luciferase reporter gene assay demonstrated an inhibitory phenomenon for mutP2 (Fig. 2B).

### HEV ORF3 affects NF-κB signaling via the TLR3 pathway

**Figure 3:**
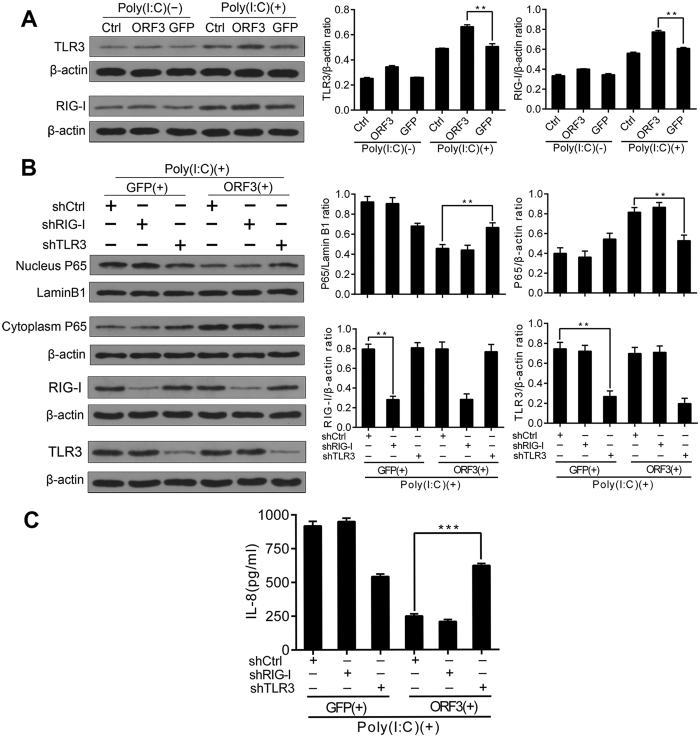
ORF3 suppresses NF-κB signaling via TLR3 signaling rather than RIG-I signaling. (**A**) The levels of TLR3 and RIG-I expression are augmented in the GFP group and in the ORF3 group with Poly(I:C). The densitometric analysis of bands is represented on the bar graph (**P < 0.01). (**B**) shTLR3 and shRIG-I were applied for gene silencing. shRNAs were co-transfected with GFP or ORF3 into cells stimulated with Poly(I:C). Densitometric analyses of the bands are represented on the bar graph. In the GFP group, P65 was translocated to the nucleus with shCtrl (control) or shRIG-I, and P65 remained in the cytoplasm with shTLR3. In the ORF3 group, most P65 protein was found in the cytoplasm with shCtrl or shRIG-I, whereas most P65 migrated to the nucleus in the shTLR3 group (**P < 0.01). Lamin B1 and β-actin served as loading controls. (**C**) The concentration of IL-8 was also used for verification. The data are representative of three independent experiments.

In addition to activating TLR3 signaling, Poly(I:C) also induces activation of the RIG-I signaling. Enhanced expression of TLR3 and RIG-I was observed in the GFP groups and in the ORF3 groups with Poly(I:C) (Fig. 3A), as verified by their mRNA levels. To exclude the influence of the two signals on each other, we applied shTLR3 and shRIG-I for gene silencing. shRNAs were co-transfected with either GFP or ORF3, and Poly(I:C) was then added to each sample. In the GFP group, P65 was translocated to the nucleus with control shCtrl or shRIG-I, but P65 remained in the cytoplasm with shTLR3; in the ORF3 group, most of the P65 resided in the cytoplasm along with the control shCtrl or shRIG-I (P > 0.05). In contrast, most of the P65 was found in the nucleus in the shTLR3 group (P < 0.05) (Fig. 3B). Use of varying concentrations of IL-8 verified these results (Fig. 3C). These findings illustrate that HEV ORF3 influences NF-κB signaling via TLR3 signaling rather than RIG-I signaling.

### HEV ORF3 restrains TLR3-induced NF-κB signaling through TRADD and RIP1

**Figure 4:**
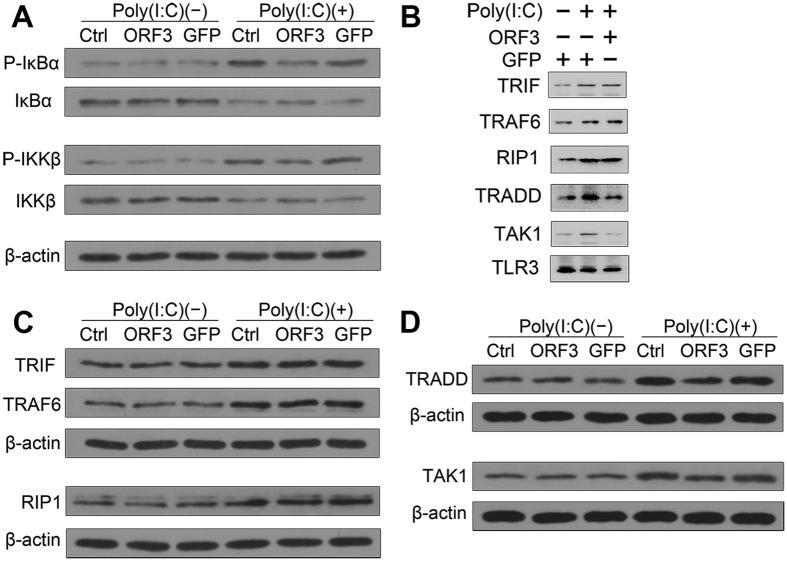
ORF3 suppresses NF-κB signaling via TRADD and RIP1. (**A**) ORF3 suppresses the phosphorylation of IKKβ and IκBα. (**B**) TLR3 pulls down of key downstream molecules detected by a coimmunoprecipitation assay. (**C,D**) TRADD and TAK1 expression levels are down-regulated in ORF3-pretreated cells compared with GFP-pretreated cells after stimulation with Poly(I:C). In contrast, TRIF, TRAF6 and RIP1 expression levels are increased by this stimulation.

To explore the inhibitory mechanism of ORF3, key downstream components of TLR3 signaling were examined. Relative to the GFP group, the ORF3 group showed reduced IκBα and IKKβ phosphorylation in response to Poly(I:C) stimulation (Fig. 4A). In addition, key downstream molecules were pulled down by TLR3 in a coimmunoprecipitation assay (Fig. 4B). Although TRIF and TRAF6 expression was up-regulated in ORF3 or GFP cells treated with Poly(I:C) (Fig. 4C), TRADD expression was down-regulated in ORF3 cells compared to GFP cells after stimulation with Poly(I:C) (Fig. 4D). Notably, RIP1 expression increased under the same conditions (Fig. 4C). The variable level of TRADD mRNA also validated the observations that ORF3 constrained Poly(I:C)-induced NF-κB activation via TRADD (data not shown) as well as the subsequent decrease in its downstream components (Fig. 4D).

**Figure 5:**
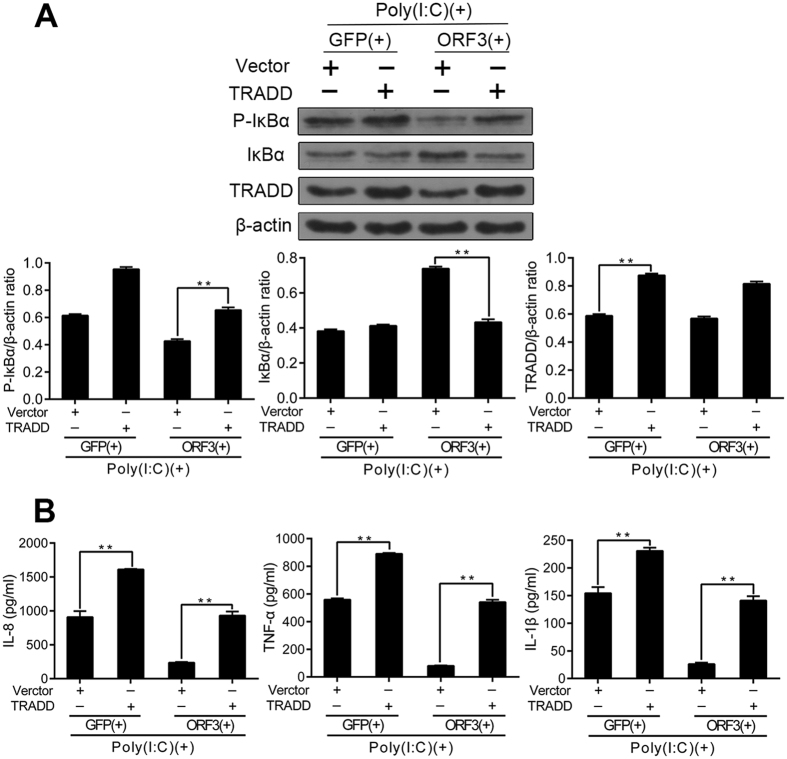
The overexpression of TRADD restores ORF3-inhibited NF-kB signaling. (**A**) TRADD plasmids were co-transfected with ORF3 at a ratio of 1:2 for 48 h, and the cells were then treated with Poly(I:C) for 12 h. Phosphorylated IκBα was assessed to measure NF-κB activity using western blotting, with β-actin serving as a loading control. Overexpression of TRADD restores ORF3-inhibited NF-κB signaling; the relative densitometric ratios for experimental bands are shown (**P < 0.01). (**B**) Poly(I:C) increases the concentration of inflammatory factors (IL-8, TNF-α and IL-1β) in the vector or TRADD groups. In presence of Poly(I:C) and ORF3, the concentration of inflammatory factors in the supernatants was lower in the vector group compared to the TRADD group (**P < 0.01). The data are given as the means ±SD representative of three independent experiments.

To further study the inhibitory mechanism of ORF3, we designed plasmids overexpressing TRADD, RIP1 or mutant RIP1 to clarify the steps of TLR3 signaling. These plasmids were co-transfected at a ratio of 1:2 with ORF3; after 48 h, the cells were stimulated with Poly(I:C) for 12 h. We then assayed phosphorylated IκBα using western blotting to measure NF-κB activity. TRADD overexpression restored ORF3-inhibited NF-κB signaling. The relative densitometric ratios for the experimental bands are presented in Fig. 5A. According to ELISA, Poly(I:C) increased the concentration of inflammatory factors (IL-8, TNF-α and IL-1β) in the vector and TRADD groups; however, the concentrations of inflammatory factors were lower in the vector group compared to the TRADD group in the presence of both Poly(I:C) and ORF3 (P < 0.01) (Fig. 5B).

### The K377R mutation of RIP1 abolishes the TRADD-mediated restoration of NF-κB activation

**Figure 6:**
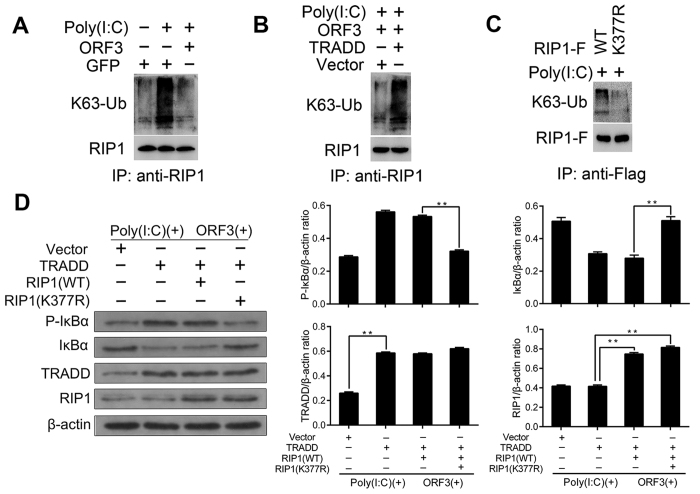
K377R mutation of RIP1 abolishes the TRADD-mediated restoration of NF-κB activation. (**A**) Pretreated cells were harvested for immunoprecipitation with an anti-RIP1 antibody to pull down K63-Ub. ORF3 inhibits K63-Ub of RIP1. (**B**) Overexpression of TRADD restores RIP1 K63-Ub. (**C**) K63-Ub is absent from K377R-mutated RIP1. (D) TRADD, RIP1 and mutant RIP1 plasmids were co-transfected with ORF3 at a ratio of 1:2 for 48 h, and the cells were then treat with Poly(I:C) for 12 h. The expression of phosphorylated IκBα was assessed to measure NF-κB activity using western blotting, with β-actin as a loading control. In the presence of Poly(I:C) and ORF3, K377R- mutated RIP1 decreased the level of phosphorylated IκBα, thus abolishing the NF-κB activation restored by TRADD (**P < 0.01). The relative densitometric ratios for experimental bands are shown.

In response to Poly(I:C) stimulation, we were able to pull down RIP1 that had undergone K63-ubiquitination (K63-Ub). However, K63-Ub of RIP1 was significantly reduced in the ORF3 group with Poly(I:C) (Fig. 6A). Interestingly, overexpression of TRADD restored RIP1 K63-Ub (Fig. 6B). We also employed RIP1 with a mutation at Lys^377^ to identify the functional ubiquitination site and found that this mutation abolished RIP1 K63-Ub (Fig. 6C). In the presence of both Poly(I:C) and ORF3, the K377R mutation of RIP1 decreased the level of IκBα phosphorylated, thus abolishing TRADD-mediated NF-κB activation. The relative densitometric ratios for the experimental bands are presented in Fig. 6D. Taken together, these data indicate that Lys^377^ may be the functional ubiquitination site of RIP1 and may play a crucial role in the TLR3-mediated NF-κB activation suppressed by ORF3.

## Discussion

Hepatitis E has become a worldwide health issue due to the lack of robust, credible surveillance and insufficient medical and laboratory infrastructure. Of note, large hepatitis E outbreaks in developing Asian countries are caused by HEV genotype 1^11^,^12^, which exhibits higher viremia and causes more severe clinical events than other genotypes^29^. Despite its association with high morbidity and mortality, this HEV genotype has received little scientific attention or research subsidization. It has been suggested that the pathogenic mechanism of HE is strongly immune mediated and does not occur via direct viral replication^30^. This leads to acute and chronic infection with a range of extra-hepatic symptoms^31^ that may be associated with altered immune states^32^. To prevent viral infection, innate immune responses are triggered in the host by PRRs, especially TLRs and RLRs. TLR3, TLR7 (TLR8) and TLR9 are residents of the ER, where they recognize extracellular viral nucleic acid species that have been endocytosed^19^. In contrast, RIG-I recognizes cytoplasmic RNA viruses via a mitochondrial adaptor protein^33^. Ultimately, PRRs elicit signaling that leads to the production of inflammatory cytokines and type I interferon^23^, which are beneficial for virus elimination.

We applied different agonists of TLRs and found that ORF3 protein suppressed Poly(I:C)-induced NF-κB activation. An anti-P65 antibody was utilized to detect the cytoplasmic and nuclear fractions of P65, and an abundance of P65 was found in the cytoplasm in ORF3-transfected cells. NF-κB has wide transcriptional regulatory functions in response to viral infection that may be altered by HEV^34^. ORF3 can interact with various cellular proteins in several clotting-related pathways, which indicates its potential role in establishing a relatively favorable environment for viral replication^9^. ORF3-expressing cells exhibit attenuated mitochondrial death signaling to survive via the up-regulation of mitochondrial voltage-dependent anion channels (VDACs) and hexokinase I^35^. The ORF3 protein increases the activity of extracellular signal-regulated kinase (ERK), which activates the MAPK cascade, resulting in the promotion of cell survival^36^. In addition, reduced levels of signal transducers and activator of transcription 3 protein (pSTAT3) have been found in ORF3-expressing cells stimulated with epidermal growth factor (EGF), leading to a reduced acute-phase response for cell survival^8^. These findings demonstrate that ORF3 has an effect on the host immune response by interacting with multiple cellular proteins, thereby optimizing the cellular environment in favor of HEV pathogenesis in an infected host.

Experimental results demonstrate that the P2 domain of ORF3 plays a suppressive role in Poly(I:C)-induced NF-κB signaling. Although the suppression mechanism remains elusive, the P2 domain does contain proline-rich sequences and two PXXP motifs, which have been delineated in some viral and cellular signaling proteins and involved in HEV budding^37^.

Poly(I:C), which mimics viral infection, is recognized by TLR3 and RIG-I^16^. Our studies showed that ORF3 protein suppressed NF-κB signaling via TLR3 signaling rather than RIG-I signaling. TLR3 is implicated in the recognition of genomic RNA purified from dsRNA viruses (including reoviruses and Poly(I:C)), dsRNA produced during the replication of ssRNA viruses (e.g., encephalomyocarditis virus [EMCV], respiratory syncytial virus [RSV], and influenza A virus [IAV])^19^,^38^; dsRNA produced as replication intermediates; ssRNA with wide-ranging secondary structures; and loopback dsRNA defective genomes acting as potential roots of dsRNA^33^. ORF3, which is derived from an ssRNA virus, generates a dsRNA intermediate that is detected by TLR3. The activation of diverse signaling pathways by TLR3 versus TLR7 (TLR8) may depend on the type of virus and TLR signaling in different cell types.

Signaling through TLR3-TRIF triggers NF-κB via two separate pathways. TRIF is the downstream adaptor molecule that serves as the platform for recruiting multiprotein signaling complexes. TRIF interacts with TRAF6 and RIP1 at distinct regions, and these molecules facilitate TAK1 activation. TAK1 phosphorylates IKKβ to increase its enzymatic activity^39^; phosphorylated IKKβ then catalyzes the phosphorylation of IκBα, ultimately resulting in NF-κB activation^40^,^41^. Initially, we found that ORF3 protein suppresses the activation of IKKβ and IκBα. Genetic research has shown that IKKβ, and not IKKα, plays an essential role in TLR-induced activation of NF-κB^26^. To further determine the inhibitory mechanism of ORF3, we continued to evaluate changes in the upstream molecules. The most intriguing results were that ORF3 suppresses NF-κB signaling via TRADD and RIP1. TRADD was originally identified more than two decades ago as an adaptor protein that interacts with the intracellular domain of TNF receptor 1^42^, leading to increases in caspase activation and cell death^43^. Reports published since then have confirmed that TRADD is necessary for the TRIF-dependent pathway and recruits ubiquitin ligases to facilitate Poly(I:C)-mediated RIP1 polyubiquitination^44^. Furthermore, it has been demonstrated that TRADD deficiency considerably impairs Poly(I:C)-induced RIP1 ubiquitination and NF-κB activation^44^. This finding is strongly consistent with our experiments, whereby ORF3 reduced TRADD expression, and impaired RIP1 ubiquitination, eventually suppressing NF-κB signaling cascades. We also observed that TRADD overexpression appeared to augment TRIF-dependent NF-κB activation. RIP1 binds to TRIF through a RIP1 homotypic interaction motif. The resulting complex contains TRADD, and complex formation is dependent on the death domain of RIP1 and TRADD^45^. Our data showed that RIP1 degradation did not occur, suggesting that RIP1 may undergo K63-ubiquitination (K63-Ub); this modification can provide docking sites for recruiting TAK1 and NEMO, which function as a scaffold for IKKβ and thus result in NF-κB activation^46^,^47^. The hallmark of Poly(I:C)-mediated IKK activation is the ubiquitination of RIP1^41^. Extensive studies have established that as an E3 ubiquitin ligase, TRAF6 may be important for MyD88-dependent signaling^48^,^49^. In contrast to TRAF6, Peli1 plays a crucial role in the ubiquitination of RIP1 at Lys63 in the TRIF-induced IKK-NF-κB signaling axis^50^,^51^. Our aforementioned findings indicated that ORF3 protein suppresses NF-κB activation via the TRADD-RIP1 pathway rather than the TRAF6 pathway. Previous studies have revealed that RIP1 has five Lys residues (Lys 305, 306, 377, 396 and 530) that are conserved among various species, with Lys^377^ being the functional ubiquitination site for triggering TNF-α induced NF-κB activation^52^. Our studies demonstrated that ORF3 decreased K63-Ub of RIP1, curbing NF-κB activation; in contrast, overexpression of TRADD restored NF-κB activation, and the K377R mutation in RIP1, prevented this restorative effect of TRADD on NF-κB activation. In summary, we demonstrate that Lys^377^ of RIP1 is a crucial ubiquitination site that is responsible for the inhibitory role of ORF3 during the Poly(I:C)-mediated activation of NF-κB. We hypothesize that K377R of RIP1 directly disrupts the association of RIP1 with TAK1^52^, leading to a loss of signal transduction.

TLRs are involved in viral replication and the host response, ultimately influencing virus pathogenesis. In this study, HEV ORF3 was found to have acquired elaborate strategies to evade host innate defense responses downstream of TLR3. These evasion strategies include degradation of the TLR3 signaling component (TRADD), deubiquitination of the signaling molecule (RIP1) and disruption of NF-κB transcriptional activity. This strategy can effectively degrade the component that relays viral association in TLR signaling and may be related to degradation of intracellular protein scaffolds by virally encoded proteases or inactivation of the host proteasome degradation system. Ubiquitination, especially K63-Ub, has an important role in activating TLR signaling pathways^53^, and deubiquitination can prevent this signal transduction. Finally, NF-κB activation can be inhibited. In terms of inflammation, NF-κB activation is the pivotal step determining cell fate by balancing survival and death signaling. ORF3 diminishes the progression of inflammation by suppressing NF-κB activation, thus facilitating cell survival.

**Figure 7:**
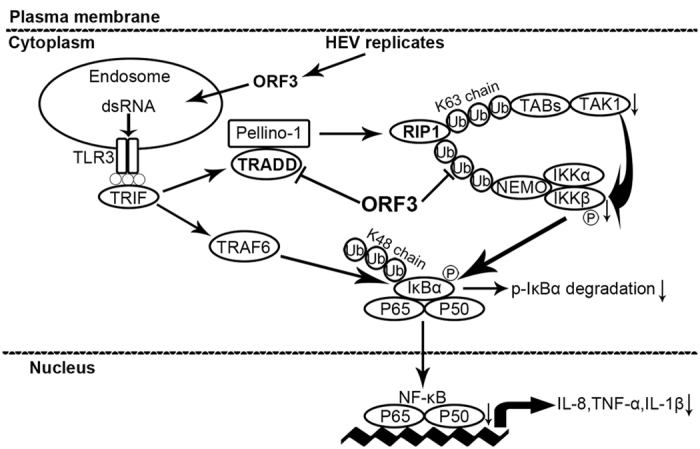
A model of the inhibitory mechanism by ORF3. We speculate that ORF3 degrades TRADD, decreases RIP1 K63-ubiquitination, and then reduces expression of downstream components, ultimately resulting in ORF3-mediated repression of TLR3-induced NF-κB signaling.

Although an effective recombinant vaccine for HEV is available in China^54^, the pathogenic mechanism of HE is poorly understood. In this paper, we provide a new explanation for how HEV ORF3 circumvents host innate immunity to facilitate virus replication. Notably, our investigation did not probe the negative regulatory effects on TRADD to explain the inhibitory effects of ORF3. Although few reports of this phenomenon exist, we will further elucidate this effect in subsequent studies. Due to the absence of effective animal models, understanding how ORF3 plays a role *in vivo* remains a challenge for future studies. In addition, the main limitation for studying HEV infection is the use of A549 cells, as these cells are not natural target cells. We found that ORF3 protein also inhibited the Poly(I:C)-induced nuclear translocation of P65 in PLC/PRF/5 cells (Figure S4), and further studies are needed to identify the mechanism of action. Interestingly, as indicated in Fig. 7, we found that TRADD and RIP1 are crucial players in HEV infection. These findings will contribute to the development of new therapeutic strategies aimed at combating HEV infection and will improve perspectives on the proper management of HEV infection.

## Materials and Methods

### Reagents and Plasmids

Polyinosinic-polycytidylic acid (Poly(I:C), TLR3 ligand), Gardiquimod (TLR7 ligand), CL075 (TLR8 ligand) and ODN2006 (TLR9 ligand) were obtained from Invivogen (San Diego, CA, USA). Antibodies specific for TLR3 (Catalogue # sc-32232, 1:300 dilution), TLR7 (# sc-57463, 1:300 dilution), TLR8 (# sc-373760, 1:300 dilution), TLR9 (# sc-52966, 1:400 dilution) and phospho-IκBα (# sc-8404, 1:400 dilution) were purchased from Santa Cruz Biotechnology (Santa Cruz, CA, USA). Anti-phospho-IKKβ (# ab59195, 1:1000 dilution), anti-ubiquitin (# ab7780, 1:1000 dilution) and linkage-specific K63 anti-ubiquitin (# ab179434, 1:1000 dilution) antibodies were obtained from Abcam (Cambridge, MA, USA). Primary antibodies against TRADD (# 3694, 1:1000 dilution), TRIF (# 4596, 1:1000 dilution), TRAF6 (# 8028, 1:800 dilution) and TAK1 (# 5206, 1:800 dilution) were obtained from Cell Signaling Technology (Beverly, MA, USA). Primary antibodies against P65 (RELA) (# 10745-1-AP, 1:1000 dilution), RIP1 (# 17519-1-AP, 1:300 dilution), RIG-I (DDX58) (# 20566-1-AP, 1:2000 dilution), IκBα (# 10268-1-AP, 1:600 dilution), IKKβ (IKBKB) (# 15649-1-AP, 1:500 dilution) and LaminB1 (# 66095-1-Ig, 1:1000 dilution) were obtained from Proteintech (Rosemont, IL, USA). Primary antibody against β-actin (# BM0627, 1:400 dilution) was purchased from Boster (Wuhan, China). A commercial anti-HEV ORF3-specific antibody (# AbM59541-29-PU, 1:1000 dilution) was obtained from Beijing Protein Innovation (Beijing, China). An anti-HEV ORF2-specific antibody (1:1000 dilution) was a gift from the National Institute of Diagnostics and Vaccine Development in infectious diseases (NIDVD, Xiamen, China).

We constructed a plasmid expressing ORF3-EGFP (ORF3) as well as plasmids lacking the D1 domain (ΔD1) and D2 domain (ΔD2) and plasmids expressing P1 domain (mutP1) and P2 domain (mutP2) mutants. All mutants were derived from the ORF3 plasmid: amino acids 15–31 were deleted in the ΔD1 plasmid and amino acids 37–62 were deleted in the ΔD2 plasmid; the mutP1 and mutP2 plasmids were generated by mutating proline to alanine. Putative TRADD and wild-type RIP1 were PCR amplified and cloned into the pLV-N-Flag/HA-XM vector. Lys^377^ of RIP1 was changed to arginine, and RIP1 (K377R) was constructed by insertion into the pLV-N-Flag/HA-XM vector.

### Cell Cultures and Transfection

Cell lines were obtained from ATCC (Manassas, VA, USA). A549 cells were cultured in Dulbecco’s modified Eagle’s medium supplemented with 10% fetal bovine serum (Gibco, Thermo Scientific, Waltham, MA, USA), penicillin (100 U/ml) and streptomycin (100 μg/ml) at 37 °C with 5% CO_2_. PLC/PRF/5 cells (hepatoma cells) were propagated in minimum essential medium under the conditions described above. The cells were transfected with plasmids expressing ORF3 or GFP with Turbofect transfection reagent (Thermo Scientific) for 2 days according to an optimized protocol. Poly(I:C) (10 μg/ml) was then added to the transfected cells to induce NF-κB activity. After another 12 h of incubation, the cells were harvested and used in experiments.

### HEV Infectious cDNA Clone and Transfection

The pSK-Sar55 cDNA clone (genotype 1 of HEV) was a kind gift from Dr. S.U. Emerson (NIH, USA). The ATG start codon of ORF3 in pSK-Sar55 was altered to GTG, which led to the loss of ORF3 protein expression. This mutant was cloned into the pSK vector by digestion with BamHI and EcoRI to construct the ΔORF3 cDNA clone. The cDNA clones were linearized with BglII, and the genomic RNA was transcribed using the MEGAscript Kit (Ambion, Thermo Scientific). A549 cells in six-well plates were transfected with RNA transcripts of pSK-Sar55 and its mutant using the DMRIE-C reagent (Invitrogen, Grand Island, NY, USA) according to the manufacturer’s protocol. The ratio of RNA to DMRIE-C was 2 μg: 8 μl. Every other day, half of the growth medium was replaced with fresh medium. After 7 days, we collected the supernatant of the cultured cells to detect the level of HEV antigen using the Diagnostic Kit for Hepatitis E Virus Antigen (Wantai, Beijing, China). To determine the establishment of HEV infection in A549 cells, a western blot analysis was performed using antibodies against ORF2 and ORF3.

### Small Hairpin RNA (shRNA)-mediated Knockdown

Using the pLV-H1-EF1a-Bsd vector (Biosettia, San Diego, CA, USA), we generated lentiviruses expressing shRNAs targeting TLR3, RIG-I, and GFP. HEK293 cells were transfected with these shRNAs using packaging plasmids (REV, VAVG, and PRRE). The HEK239 cell supernatants containing lentiviruses were harvested at 48–60 h post-transfection and then inoculated into A549 cells. After 48 h, the cells were collected for western blot analysis to test the efficiency of interference. The target sequences for human TLR3, RIG-I and GFP were 5′-AAAATCACGCAATTGGAAGATTATTGGATCCAATAATCTTCCAATTGCGTGA-3′, 5′-AAAAGCCAGAATCTTAGTGAGAATTTTGGATCCAAAATTCTCACTAAGATTCTGGC-3′ and 5′-TACAACAGCCACAACGTCTAT-3′, respectively.

### Immunoprecipitation

Cells were washed with cold PBS and lysed with lysis buffer (50 mM Tris-HCl, pH 8.0, 150 mM NaCl, 0.5% deoxycholate acid, 1% NP-40) containing phosphatase and protease inhibitors. For immunoprecipitation, the lysates were incubated with the indicated primary antibodies or anti-Flag M2 affinity resin (Sigma Aldrich, MO, USA) at 4 °C overnight and then further incubated with protein A-agarose (Millipore, Billerica, MA, USA) for 1 h at 4 °C with gentle shaking. After five washes with lysis buffer, the immunoprecipitated proteins were boiled with 2 × SDS loading buffer, separated by SDS-polyacrylamide gel electrophoresis (PAGE) and electrophoretically transferred to polyvinylidene difluoride (PVDF) membranes (Millipore). The membranes were blocked with 5% BSA in Tris-buffered saline containing 0.1% Tween-20 and immunoreacted with the indicated primary antibodies and HRP-conjugated secondary antibodies.

### Western Blot Analysis

Cells were lysed in RIPA buffer (Beyotime Biotechnology, Shanghai, China) containing 50 mM Tris (pH 7.4), 150 mM NaCl, 1% Triton X-100, 1% sodium deoxycholate, 1% SDS and EDTA. Protein concentrations were measured using an Enhanced BCA Protein Assay Kit (Beyotime). Cytoplasmic and Nuclear Protein Extraction kits (Boster, Wuhan, China) were used to prepare nuclear and cytoplasmic extracts. Equal amounts of proteins (50 μg) were subjected to SDS-PAGE and transferred to PVDF membranes. The membranes were blocked with 5% skim milk (diluted in TBST) for 2 h, and then incubated overnight with specific primary antibodies. After washing with TBST containing 0.1% Tween 20, the membranes were subsequently incubated with ECL horseradish peroxidase-linked secondary antibodies. The PVDF membranes were exposed to film, and the protein bands were visualized using a scanner system and densitometric analysis with Bandscan software. The relative densitometric ratio of the experimental bands was calculated by normalization using a loading control (LaminB1 or β-actin).

### Immunofluorescence Assay

For immunofluorescence staining and colocalization experiments, A549 cells were seeded on coverslips in 24-well plates and grown to 60–80% confluence. The cells were transfected with either the GFP or ORF3 plasmid; at 48 h post-transfection, the cells were treated with 10 μg/ml Poly(I:C) for 12 h. The cells were washed briefly in PBS, fixed in 4% pre-cold paraformaldehyde for 15 min in a dark room at room temperature, and permeabilized in 0.3% Triton X-100 for 20 min. The cells were blocked in pure goat serum for 1 h at room temperature. P65 was detected using a rabbit anti-human P65 antibody (1:50 dilution), followed by Cy3-coupled goat anti-rabbit IgG (1:100 dilution). The nuclei were counterstained with 4′-6′-diamidino-2-phenylindole (DAPI, 1:1000 dilution). The subcellular localization of P65 (red) with GFP or ORF3 (green) was observed with an Olympus BX53 fluorescence microscope (Japan).

### Real-time Reverse Transcriptase-Polymerase Chain Reaction (RT-PCR)

Total RNA from A549 cells was extracted with Trizol (Thermo Scientific). We identified 42 genes associated with key downstream molecules and inflammatory cytokines in TLR3 signaling and RIG-I signaling. All RNA samples were reverse transcribed to cDNA using TaqMan Reverse Transcription Reagents (Thermo Scientific). The mRNA levels of ACTB, B2M, OAZ1, PPC, GDC and GAPDH (housekeeping control) were quantified by real-time PCR using SYBR master mix (Thermo Scientific). Gene-specific primer sequences were obtained from BGI (Shenzhen China). The PCR amplification conditions included 1 cycle of 95 °C for 2 min and, 40 cycles of 95 °C for 10 s, and 60 °C for 20 s; a Roche Light Cycler 480 II Real-Time PCR System was used. The threshold cycle (CT) values were corrected using the average values of the housekeeping genes. The mRNA abundance of each indicated gene was calculated using the formula 2^−ΔΔCt^, and the results are expressed as fold changes.

### Enzyme-linked Immunosorbent Assay (ELISA)

The concentrations of NF-κB-responsive genes (IL-8, TNF-a and IL-1β) were evaluated in cell culture supernatants according to the manufacturer’s protocol (Joyee Biotechnics, Shanghai, China). All samples were tested in triplicate.

### The Dual-luciferase Reporter Gene Assay

A549 cells were seeded in a 12-well plate and grown overnight. The cells were then co-transfected with 20 ng of pNF-κB-Luc (firefly luciferase) (Beyotime) and 5 ng of pRL-TK (Renilla luciferase) (Promega, Madison, WI, USA) with the indicated amounts of expression plasmids (GFP, ORF3, ΔD1, ΔD2, mutP1, and mutP2). After 48 h, the cells were stimulated (or not) with Poly(I:C) for an additional 12 h. Cell lysates were prepared and assayed for luciferase activity using a Dual-Luciferase Assay Kit (Promega). To correct for transfection efficiency, the firefly luciferase values were normalized based on the Renilla luciferase values. The relative fold changes in luciferase activity compared with the control cells were quantified as shown.

### Statistical Analysis

All experiments were performed at least three times. The values are presented as the means ± standard deviation (SD) for normally distributed data. Statistical analysis was performed on the ELISA and WB results using GraphPad Prism software. Statistical comparisons were made using unpaired Student’s *t*-tests. All hypotheses were assessed with two-tailed P values, and values less than 0.05 were considered statistically significant.

## Additional Information

**How to cite this article**: He, M. *et al.* The ORF3 Protein of Genotype 1 Hepatitis E Virus Suppresses TLR3-induced NF-κB Signaling via TRADD and RIP1. *Sci. Rep.* **6**, 27597; doi: 10.1038/srep27597 (2016).

## Supplementary Material

Supplementary Information
