# Supplementary material for: The ORF3 Protein of Genotype 1 Hepatitis E Virus Suppresses TLR3-induced NF-κB Signaling via TRADD and RIP1
Source: Sci Rep. 2016 Jun 8;6:27597. doi: 10.1038/srep27597 (PMC4897786; doi:10.1038/srep27597)
Supplement: Supplementary Information [file srep27597-s1.pdf]

The ORF3 Protein of Genotype 1 Hepatitis E Virus Suppresses TLR3-induced NF-κB Signaling via TRADD and RIP1

Man He[1], Min Wang[1], Ying Huang[1], Wenju Peng[1], Zizheng Zheng[2], Ningshao Xia[2], Jian Xu[3], Deying Tian[1]*

[1] Department of Gastroenterology, Tongji Hospital, Tongji Medical College, Huazhong University of Science and Technology, Wuhan, 430030, China. [2] National Institute of Diagnostics and Vaccine Development in Infectious Disease, School of Public Health, Xiamen University, Xiamen, 361005, China. [3] Department of Infectious Disease, the Central Hospital of Fuling District, Chongqing, 404100, China.

*Address correspondence to Dr. Deying Tian, dytian@tjh.tjmu.edu.cn

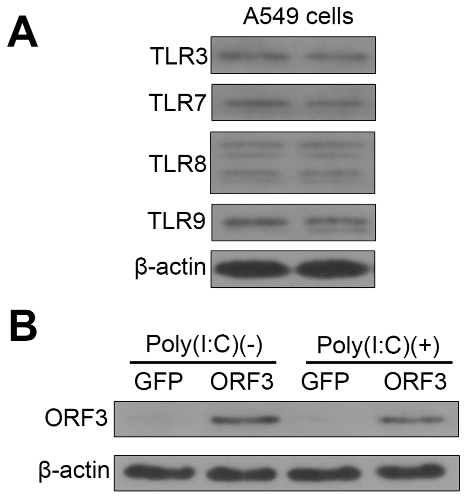

Figure S1. (A) TLR3, 7, 8, and 9 expression in A549 cells. (B) HEV ORF3 expresses in A549 cells.

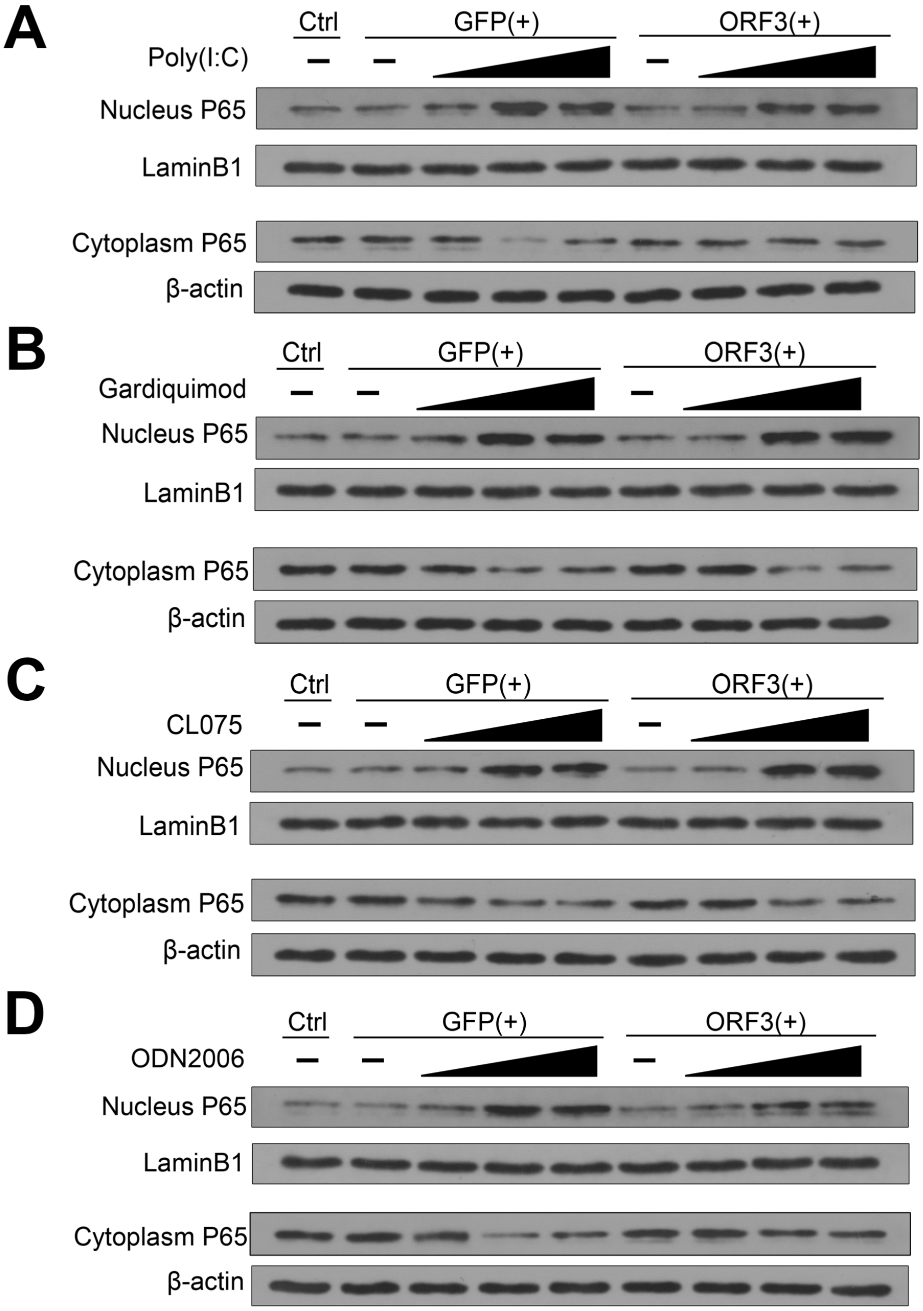

Figure S2. The dose-dependent assay of the TLR agonist identified P65 in the nucleus and cytoplasm extracts. (A-D) Poly(I:C) (TLR3 ligand) (1μg/ml, 10μg/ml, 20μg/ml), Gardiquimod (TLR7 ligand) (1μg/ml, 3μg/ml, 5μg/ml), CL075 (TLR8 ligand) (1μg/ml, 5μg/ml, 10μg/ml) and ODN2006 (TLR9 ligand) (1 μM, 3 μM, 5 μM) had peak effects on NF-κB signaling at concentrations of 10 μg/ml, 3μg/ml, 5μg/ml, and 3 μM, respectively.

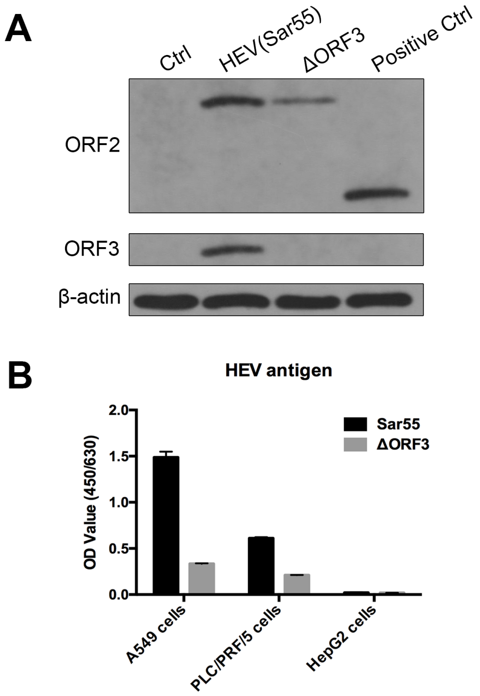

Figure S3. (A) A549 cells were successfully infected with Sar55 and ΔORF3. (B) With the exception of the HepG2 cells, all other cells were successfully cultured with the infectious clones of Sar55 and ΔORF3.

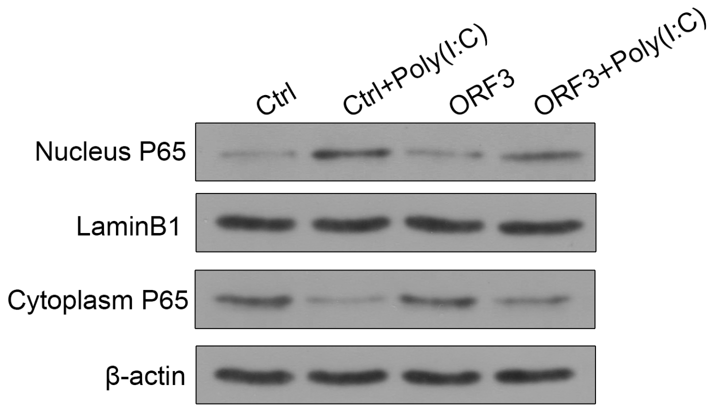

Figure S4. HEV ORF3 also inhibites the Poly(I:C)-induced nuclear translocation of P65 in PLC/PRF/5 cells.
